# Association between geriatric nutritional risk index and thoracic aorta morphological changes in cancer adults underwent contrast computed tomography scans

**DOI:** 10.3389/fnut.2025.1583220

**Published:** 2025-08-22

**Authors:** Zhijie Jian, Zixuan Meng, Xiangrui Qiao, Hui Liu, Bolin Li, Yue Wu, Wenjun Liu, Lele Cheng

**Affiliations:** ^1^Department of Medical Imaging, The First Affiliated Hospital of Xi'an Jiaotong University, Xi'an, China; ^2^Department of Cardiovascular Medicine, The First Affiliated Hospital of Xi'an Jiaotong University, Xi'an, China; ^3^Biobank, The First Affiliated Hospital of Xi'an Jiaotong University, Xi'an, China

**Keywords:** geriatric nutritional risk index, thoracic aorta tortuosity, cancer, computed tomography, malnutrition

## Abstract

**Background:**

Cancer survivors have a heightened risk of cardiovascular disease (CVD), partly associated with high rates of malnutrition, which is linked to poor cardiovascular outcomes. Changes in aortic morphology affect vascular hemodynamics and cardiovascular health. However, the relationship between malnutrition and aortic morphology in cancer patients remains unreported. This study aims to investigate the relationship between malnutrition and thoracic aorta morphological changes in cancer adults.

**Methods:**

We performed a cross-sectional study of 189 adults without known cardiovascular disease who underwent computed tomography (CT) enhanced scan between 2020 and 2021. All patients were divided into three groups according to three categories of the geriatric nutritional risk index (GNRI): moderate to severe, GNRI of < 92 (*n* = 54); low, GNRI of 92–98 (*n* = 36); and absence of risk, GNRI of ≥98 (*n* = 99). The morphology of the aorta was measured by segmental diameters and tortuosity using CT.

**Results:**

A total of 189 patients were included in the study. The average age in this study was 60.8 ± 16.5 years, with 115 men (60.8%). About half of the patients were at risk of malnutrition. Compared with the absent-risk group, participants with low or moderate to severe risk exhibited significantly larger diameters and more tortuosity of the ascending and arcus aorta, thoracic aorta, and descending thoracic aorta (all *P* < 0.05). We observed linear and negative associations of the GNRI value with the diameter in L1–L3 (*r* = −0.47, *r* = −0.48, *r* = −0.47, respectively; all *P* < 0.001) and tortuosity of the ascending and arcus aorta, thoracic aorta and descending thoracic aorta (*r* = −0.54, *r* = −0.53, *r* = −0.59, all *P* < 0.001). Besides, there were significant associations between malnourishment risk and morphological characteristics of the thoracic aorta in both the adjusted and unadjusted linear regression models, especially in older patients.

**Conclusions:**

Our findings indicate that malnutrition measured by GNRI is linked to aortic diameter and tortuosity in cancer patients, reflecting the exploratory role in identifying malnutrition as a novel risk marker in cardio-oncology. Future studies could explore whether improving GNRI through targeted nutritional support mitigates aortic remodeling.

## 1 Background

Cardiovascular disease (CVD) is a major cause of illness and mortality among cancer survivors (1, 2). Identifying the modifiable risk factors associated with cardiovascular changes in these individuals is crucial for improving cardiovascular outcomes. Recent studies indicate that cancer patients face a heightened risk of cardiovascular death and complications, irrespective of cancer type, even when traditional risk factors are accounted for (3, 4). Malnutrition is a prevalent complication among oncology patients, stemming from both the disease itself and its treatments, including surgery, chemotherapy, and radiation (5). Malnutrition can lead to increased systemic inflammation and oxidative stress, both of which accelerate endothelial dysfunction and vascular remodeling by promoting extracellular matrix degradation, smooth muscle apoptosis, and collagen dysregulation (6–8). These processes may directly compromise aortic wall integrity, predisposing to dilatation and tortuosity. Additionally, malnutrition impairs protein synthesis and energy metabolism, reducing the availability of substrates like albumin and amino acids critical for maintaining vascular elasticity and smooth muscle contractility (9). The resultant loss of aortic compliance may exacerbate hemodynamic stress, further driving structural remodeling. While malnutrition's role in cardiovascular mortality is established, its direct impact on aortic morphology remains unexplored in cancer survivors.

The thoracic aorta plays a vital role in transporting blood from the heart to the organs, which is essential for maintaining perfusion homeostasis (10). Changes in the aorta's shape, such as tortuosity and dilation, can disrupt its function, affecting blood flow dynamics. This can increase the heart's workload, overstretch the aortic wall, and decrease coronary blood flow. The morphological changes of the aorta can be accurately assessed using invasive angiography, such as computed tomography (CT) which exhibits high reproducibility (11, 12). During the treatment of cancer patients, enhanced CT scans are often performed to evaluate disease progression, facilitating the visualization of the aorta. This allows for the analysis of aortic morphology to monitor vascular changes in cancer patients.

Nutritional status can be evaluated using a straightforward and reliable tool known as the geriatric nutritional risk index (GNRI), which is based on three parameters: height, body weight, and serum albumin level (13). The GNRI has been reported to correlate with adverse cardiovascular outcomes in high-risk populations, including the elderly, individuals with chronic kidney disease, and those suffering from chronic cardiovascular disease (13–16). A recent study found that low GNRI and high brachial-ankle pulse wave velocity are linked to small fiber neuropathy in hemodialysis patients, indicating a possible connection between malnutrition and arterial stiffness (17). Consequently, This study aims to explore if malnutrition, measured by GNRI, is related to changes in the thoracic aorta, such as dilatation and tortuosity, in cancer survivors.

## 2 Materials and methods

### 2.1 Study population

A single-center retrospective study was conducted at the First Affiliated Hospital of Xi'an Jiaotong University. Patients with malignancies who underwent enhanced CT scans between July 2021 and October 2022 were included. Only those with malignant tumors confirmed by surgical pathology were considered. The exclusion criteria consisted of the following: (1) individuals with a prior diagnosis of hypertension, diabetes, or kidney disease; (2) individuals with aneurysms, dissections, vascular malformations, or variations observed on the CT scan; (3) poor-quality CT scans with significant artifacts, and (4) individuals lacking sufficient clinical data for analysis. Written informed consent was obtained from all study participants, and the study received approval from the ethics committee of the First Affiliated Hospital of Xi'an Jiaotong University (Approved number: XJTU1AF2020LSL-018).

### 2.2 CT image acquisition and assessment of diameters and tortuosity index of thoracic aorta

Thoracic enhanced CT scan was performed using two 256-slice CT scanners (Philips Brilliance iCT, Medical Systems, Best, The Netherlands; Revolution CT; GE Healthcare, Milwaukee, WI). Scans were obtained from the lung apices to the iliac crest, with non-ECG-gating.

**Figure 1:**
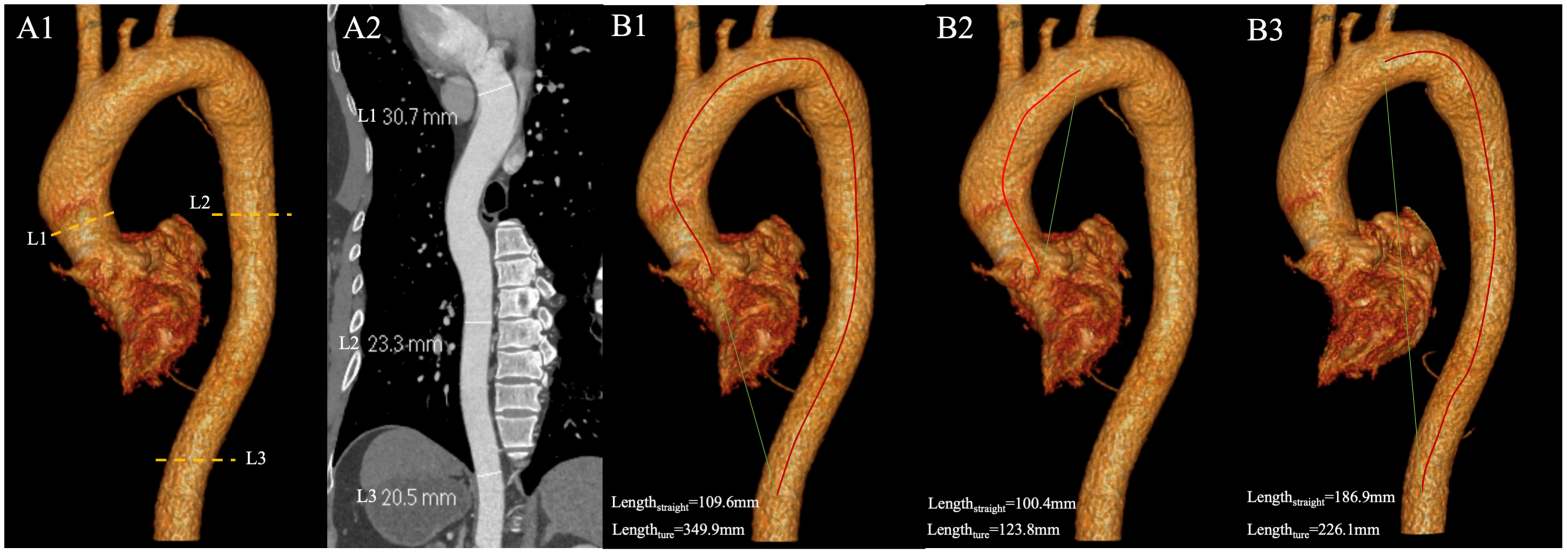
**(A1)** The diameter of the thoracic aorta were measured at three locations with enhanced CT images. L1 is located 1 cm distal to the sinotubular junction; L2 is in line with L1; L3 at the level of the diaphragm; **(A2)** Diameters of L1, L2, and L3 measured on curved planar reconstructions of the thoracic aorta; **(B)** Measurement of tortuosity index. The red line is the true length measurement of the vessel, and the green line is the straight-line length. **(B1)** The true length of the thoracic aorta is 349.9 mm, and the straight-line length is 109.6 mm. The tortuosity index of the thoracic aorta is 3.19. **(B2)** The true length of the ascending aorta and the aortic arch is 123.8 mm, and the straight-line length is 100.4 mm. The tortuosity index of the segment is 1.23. **(B3)** The true length of the DTA is 226.1 mm, and the straight-line length is 186.9 mm. The tortuosity index of DTA is 1.21. DTA, descending thoracic aorta.

All data were analyzed using a standard post-processing workstation (uInnovation-CT, R001, United Imaging Healthcare, Shanghai, China), with commercially available volume viewer software. We measured the diameter of the thoracic aorta using the outer edge-to-outer edge method at three aortic levels: the ascending aorta (L1), located 1 cm distal to the sinotubular junction; the descending aorta (L2), aligned with L1; and at the level of the diaphragm (L3; Figure 1). Subsequently, we calculated the tortuosity of the thoracic aorta, defined as extending from the aortic valve to the aortic diaphragmatic hiatus (Figure 1). An automated centerline between these two points was generated, and both the centerline length of the aorta and the straight-line length between the two points were documented. The tortuosity index of the thoracic aorta was then calculated. All imaging analyses were independently evaluated by two cardiovascular radiologists, who had 12 and 6 years of experience in cardiac imaging, respectively; the mean values of their measurements were used for further analysis. Radiologists were fully blinded to patient GNRI status, clinical outcomes, and all non-imaging data during imaging assessments. Additionally, we standardized the aorta diameter and tortuosity by body surface area (BSA) to mitigate the impact of varying body shapes on the data analysis. BSA was calculated using the Dubois and Dubois formula (18): BSA (m^2^) = 0.007184 × height (cm)^0.725^ × weight (kg)^0.425^.

### 2.3 Measurement of GNRI

The GNRI was calculated using the formula: [1.489 × albumin (g/L)] + [41.7 × (body weight/ideal body weight)] (13). The ideal body weight was determined using the formula: 22 × (height squared), due to its established validity (19, 20). If a patient's body weight exceeded their ideal body weight, the weight-to-ideal body weight ratio was set to 1 (13). These variables were assessed during the baseline visit at enrollment. Patients were categorized according to the following cutoffs: moderate to severe malnourishment risk: < 92; low risk: 92– < 98; and absence of risk: ≥98.

### 2.4 Demographic and clinical data

We collected several demographics, and clinical and analytical parameters. Age, gender, height, and weight were recorded. Peripheral blood was sampled from patients within 24 h of admission. Biochemical indicators were tested by the laboratory using standard methods.

### 2.5 Statistical analysis

Continuous variables are presented as the mean ± standard deviation if normally distributed, or as the median (lower quartile, upper quartile) otherwise. Categorical variables are reported as numbers and percentages. Differences in parameters among groups were analyzed using analysis of variance for normally distributed variables; the Kruskal–Wallis test was employed for non-normally distributed continuous variables, and the chi-square test was utilized for categorical variables. Univariate and multivariate linear regression models were employed to estimate the association between malnutrition and the L1–L3 diameter as well as the tortuosity of the thoracic aorta. To adjust for potential confounding factors, two models were established: Model 1 adjusted for age and sex, while Model 2 adjusted for age, sex, cholesterol, creatinine, hemoglobin, fasting blood glucose, indirect bilirubin, and glutamic-pyruvic transaminase. The regression coefficients and their 95% confidence intervals (CIs) are presented. Additionally, Spearman's correlation analyses of the GNRI with the morphological characteristics of the thoracic aorta were performed.

All analyses were conducted using R software (version 4.0.1), and a difference was considered statistically significant at a two-sided *P*-value of < 0.05.

## 3 Results

### 3.1 Clinical baseline characteristics of subjects

A total of 189 patients were included in the study. The average age of participants was 60.8 ± 16.5 years, comprising 74 women (39.2%) and 115 men (60.8%). Patients were categorized into three groups: absence of risk (*n* = 99), low risk (*n* = 36), and moderate to severe risk (*n* = 54). The clinical characteristics of the patients are detailed in Table 1. Compared to the absence of risk group, individuals in the moderate to severe risk groups were older, had a higher proportion of females, and exhibited lower levels of cholesterol, total protein, and albumin. Additionally, there were significant differences in levels of indirect bilirubin, alanine aminotransferase, creatinine, hemoglobin, and diastolic blood pressure among the three groups.

**Table 1:** Clinical characteristics of patients according to GNRI.

**Variables**	**Absence of risk (*n* = 99)**	**Low risk (*n* = 36)**	**Moderate/Severe risk (*n* = 54)**	***P*-value**
GNRI	104.08 (101.66–109.99)	95.61 (93.87–96.29)	83.60 (77.51–89.26)	<0.001
Age, years	58.59 ± 14.67	61.39 ± 16.62)	64.46 ± 19.24	0.020
Male, %	63 (63.64%)	28 (77.78%)	24 (44.44%)	0.005
Cholesterol, mmol/L	4.21 (3.55–4.87)	4.18 (3.57–4.67)	3.47 (2.67–4.12)	0.001
Indirect bilirubin, mmol/L	7.35 (5.00–10.78)	5.30 (4.20–8.65)	6.20 (3.87–10.18)	0.034
Total bilirubin, mmol/L	9.60 (7.23–13.17)	8.10 (6.00–11.65)	8.60 (5.70–13.15)	0.087
Total protein, g/L	64.90 (61.20–69.05)	62.20 (58.42–64.70)	56.95 (53.35–60.50)	<0.001
Aspartate transaminase, U/L	22.00 (18.00–28.00)	20.00 (16.75–23.25)	20.00 (16.00–27.00)	0.213
Alanine aminotransferase, U/L	19.00 (13.00–27.00)	13.50 (9.75–20.50)	16.50 (9.25–25.00)	0.025
Alkaline phosphatase, U/L	78.00 (64.00–100.00)	85.00 (74.50–93.00)	84.50 (63.00–111.50)	0.323
Albumin, g/L	38.90 (36.75–41.75)	36.15 (34.38–37.28)	30.85 (27.80–33.77)	<0.001
Urea, mmol/L	5.13 (4.33–6.04)	5.53 (4.50–6.29)	5.20 (3.98–6.67)	0.969
Creatinine, μmol/L	59.00 (50.00–69.50)	61.50 (51.00–69.00)	52.50 (43.25–61.00)	0.012
eGFR, mL/min	103.95 (92.28–112.73)	104.97 (85.56–113.84)	98.13 (87.05–109.04)	0.632
Hemoglobin, g/L	129.00 (118.00–143.75)	130.50 (117.75–134.25)	112.50 (96.75–125.00)	<0.001
White blood cell, 10^9^/L	5.53 (4.50–6.77)	5.96 (4.56–7.68)	6.40 (4.90–8.46)	0.043
Systolic pressure, mmHg	124.00 (113.00–136.00)	121.00 (114.25–133.25)	122.00 (111.50–135.75)	0.872
Diastolic pressure, mmHg	76.00 (72.00–80.50)	76.00 (70.75–80.25)	72.00 (67.00–78.75)	0.033
Fasting glucose, mmol/L	5.20 (4.45–6.05)	4.83 (4.36–5.52)	4.64 (4.07–5.75)	0.068
**Primary tumor site**				0.104
Lung	49(49.5%)	13(36.1%)	14(25.9%)	
Digestive system	39(39.4%)	16(44.4%)	26(48.1%)	
Blood system	4(4.0%)	2(5.6%)	5(9.3%)	
Urinary/reproductive system	1(1.0%)	3(8.3%)	3(5.6%)	
Other	6(6.1%)	2(5.6%)	6(11.1%)	

GNRI, geriatric nutritional risk index; eGFR, estimated glomerular filtration rate.

### 3.2 Morphological characteristics of the thoracic aorta in subjects

**Figure 2:**
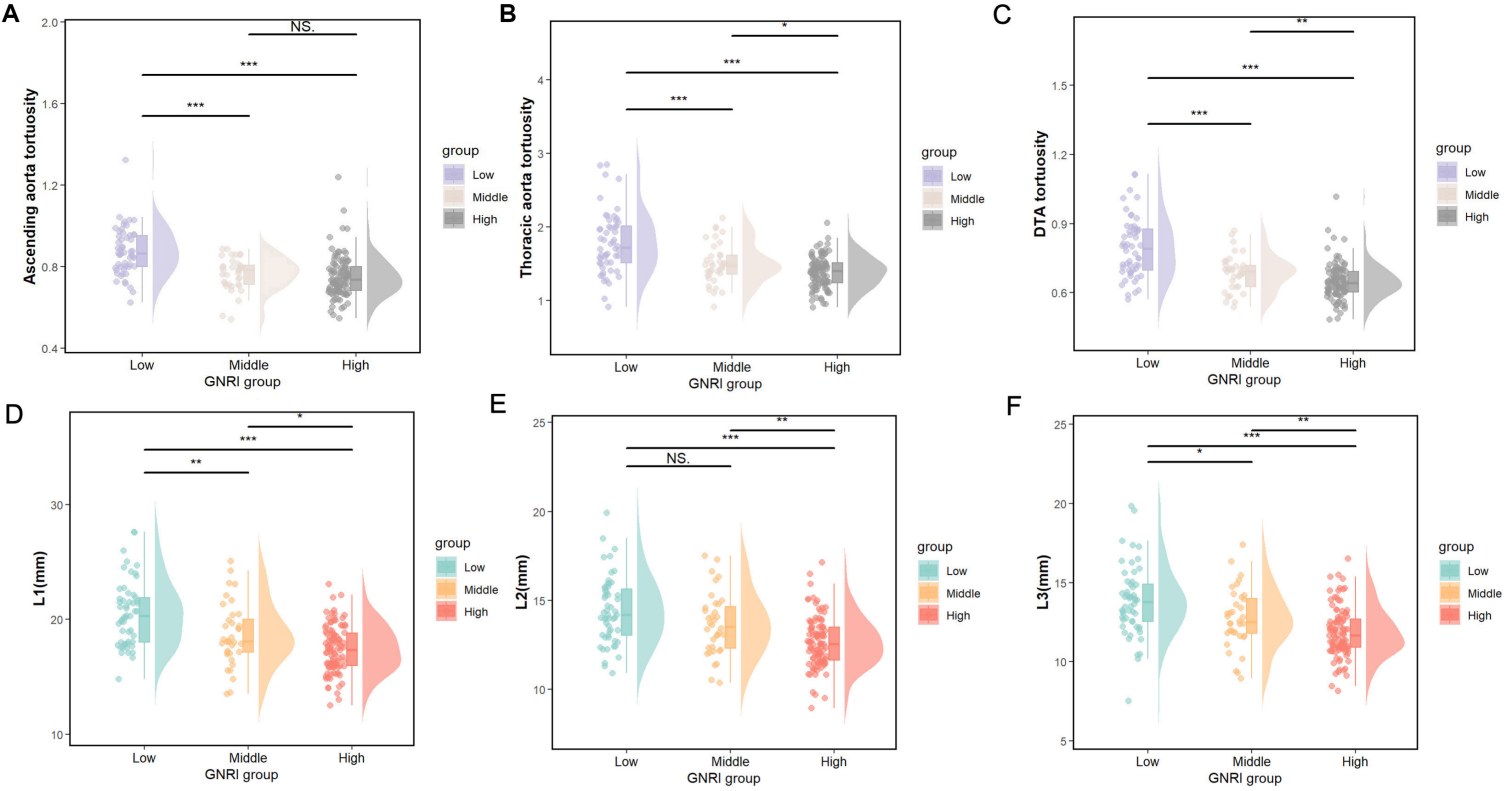
**(A–C)** The distributions of ascending aorta tortuosity, thoracic aorta tortuosity, and DTA tortuosity and **(D–F)**, the L1–L3 diameter, among the GNRI groups according to the nutritional status. Low, moderate/severe risk; Middle, low risk; High, absence of risk. ^*^p < 0.05, ^**^p < 0.01, ^***^p < 0.001, NS. *p* > 0.05.

Table 2 illustrates the relationship between the GNRI value and the morphological characteristics of the thoracic aorta in participants. Compared to the absent-risk group, participants classified as having low or moderate to severe risk exhibited significantly larger diameters in the L1–L3 region, as well as increased tortuosity of the ascending and arcus aorta, thoracic aorta, and descending thoracic aorta (DTA; all *P* < 0.05). Figure 2 presents the distributions of diameters and tortuosity of the thoracic aorta across the groups categorized by the GNRI.

**Table 2:** Imaging characteristics of patients according to GNRI.

**Variables**	**Absence of risk (*n* = 99)**	**Low risk (*n* = 36)**	**Moderate/Severe risk (*n* = 54)**	***P*-value**
Ascending and arcus aorta tortuosity	0.74 (0.68–0.80)	0.78 (0.71–0.81)	0.87 (0.80–0.95)	<0.001
Thoracic aorta tortuosity	1.41 (1.24–1.51)	1.47 (1.36–1.62)	1.73 (1.52–2.01)	<0.001
DTA tortuosity	0.64 (0.60–0.69)	0.69 (0.63–0.72)	0.79 (0.70–0.88)	<0.001
L1, mm	17.37 (16.00–18.80)	18.11 (17.13–20.04)	20.33 (18.04–21.90)	<0.001
L2, mm	12.56 (11.64–13.50)	13.53 (12.32–14.65)	14.20 (13.04–15.65)	<0.001
L3, mm	11.67 (10.90–12.70)	12.50 (11.78–13.99)	13.78 (12.55–14.88)	<0.001

DTA, Descending thoracic aorta.

### 3.3 Relationship between GNRI and the thoracic aorta morphology

**Figure 3:**
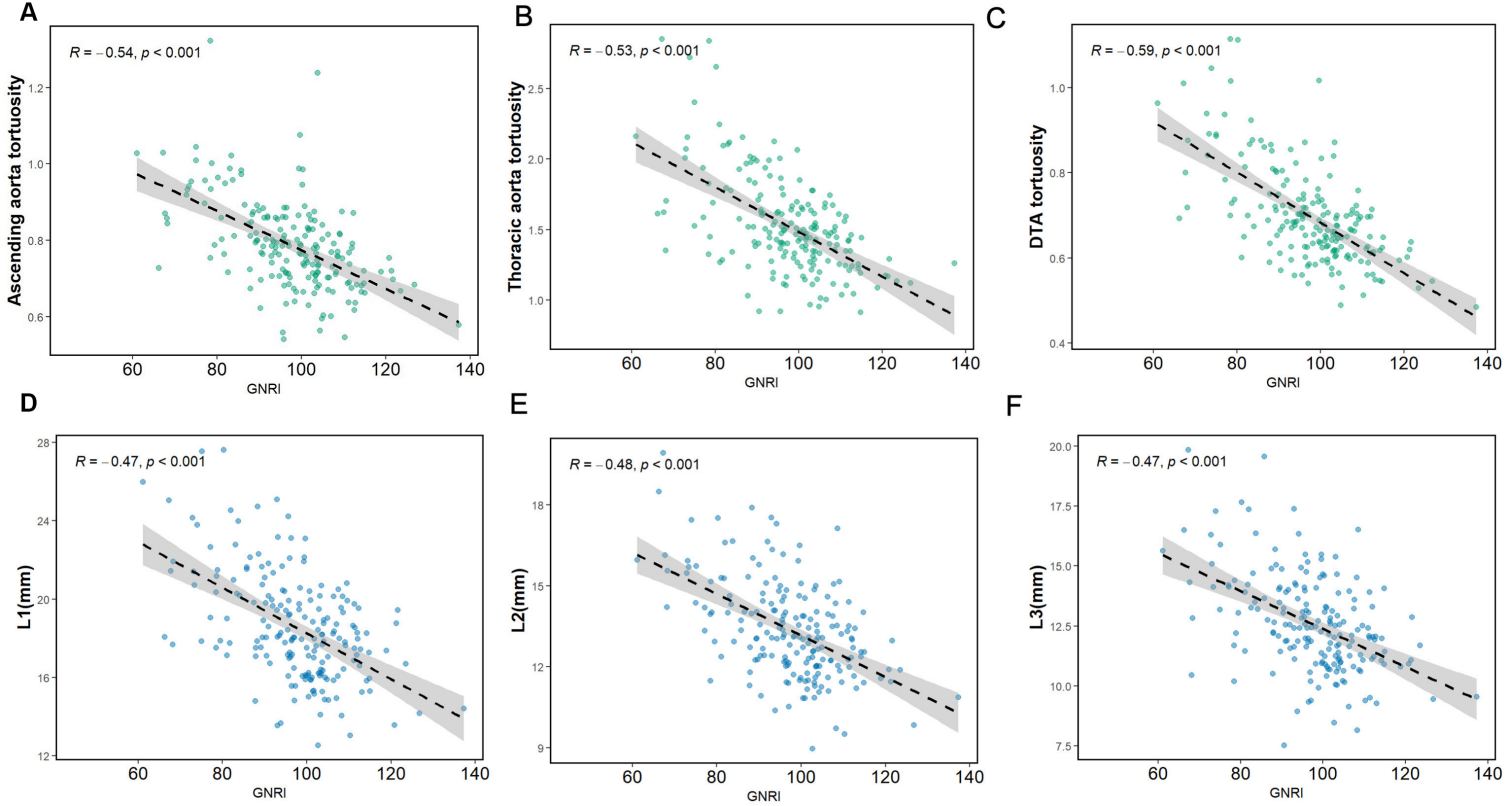
The regression line of the GNRI with the diameter and tortuosity of aorta. **(A)** GNRI and ascending aorta tortuosity. **(B)** GNRI and thoracic aorta tortuosity. **(C)** GNRI and DTA tortuosity. **(D–F)** GNRI and L1–L3 diameter.

We observed linear and negative associations between the GNRI value and the diameters at L1–L3 (*r* = −0.47, *P* < 0.001; *r* = −0.48, *P* < 0.001; *r* = −0.47, *P* < 0.001, respectively) as well as the tortuosity of the ascending and arcus aorta, thoracic aorta, and the DTA (*r* = −0.54, *P* < 0.001; *r* = −0.53, *P* < 0.001; *r* = −0.59, *P* < 0.001), as illustrated in Figure 3. The correlation coefficients of the GNRI with the morphological characteristics of the thoracic aorta are provided in Supplementary Table S1.

There were significant associations between the risk of malnourishment and the morphological characteristics of the thoracic aorta in both the adjusted and unadjusted models. In the fully adjusted model (Model 2), the average increases in L1–L3 diameter changes when comparing participants in the moderate to severe risk group to those in the absent-risk group were 2.45 mm (95% CI: 1.59–3.31, *P* < 0.001), 1.45 mm (95% CI: 0.89–2.01, *P* < 0.001), and 1.60 mm (95% CI: 1.00–2.20, *P* < 0.001), respectively. Similarly, the average tortuosity increases in ascending and arcus aorta, thoracic aorta, and DTA tortuosity for the moderate to severe risk group compared with the absent-risk group were 0.10 (95% CI: 0.06–0.13, *P* < 0.001), 0.30 (95% CI: 0.21–0.39, *P* < 0.001), and 0.09 (95% CI: 0.06–0.12, *P* < 0.001). The detailed regression results are presented in Table 3.

**Table 3:** Univariable and multivariable linear regression analysis between nutritional status and L1–L3 diameter, ascending and arcus aorta tortuosity, thoracic aorta tortuosity and DTA tortuosity.

**Statistics**	**Ascending and arcus aorta tortuosity**	**Thoracic aorta tortuosity**	**DTA tortuosity**	**L1 (mm)**	**L2 (mm)**	**L3 (mm)**
	β **(95% CI)** ***p*** **Value**	β **(95% CI)** ***p*** **Value**	β **(95% CI)** ***p*** **Value**	β **(95% CI)** ***p*** **Value**	β **(95% CI)** ***p*** **Value**	β **(95% CI)** ***p*** **Value**
**Unadjusted**						
Absence of Risk	Reference	Reference	Reference	Reference	Reference	Reference
Low Risk	0.01 (–0.03, 0.05) 0.518	0.11 (–0.00, 0.23) 0.054	0.04 (0.00, 0.07) 0.046	1.25 (0.32, 2.18) 0.009	1.00 (0.38, 1.63) 0.002	0.89 (0.18, 1.60) 0.014
Moderate/Severe Risk	0.12 (0.09, 0.16) < 0.001	0.40 (0.30, 0.50) < 0.001	0.15 (0.11, 0.18) < 0.001	3.12 (2.31, 3.92) < 0.001	1.78 (1.24, 2.32) < 0.001	1.98 (1.36, 2.59) < 0.001
**Model 1**						
Absence of Risk	Reference	Reference	Reference	Reference	Reference	Reference
Low Risk	0.03 (–0.01, 0.06) 0.137	0.11 (0.02, 0.20) 0.020	0.05 (0.02, 0.08) 0.003	1.31 (0.48, 2.14) 0.002	0.91 (0.37, 1.44) 0.001	0.78 (0.19, 1.38) 0.010
Moderate/Severe Risk	0.10 (0.07, 0.14) < 0.001	0.31 (0.23, 0.39) < 0.001	0.11 (0.09, 0.14) < 0.001	2.51 (1.77, 3.24) < 0.001	1.41 (0.93, 1.88) < 0.001	1.52 (0.99, 2.04) < 0.001
**Model 2**						
Absence of Risk	Reference	Reference	Reference	Reference	Reference	Reference
Low Risk	0.03 (–0.01, 0.07) 0.184	0.11 (0.01, 0.21) 0.030	0.04 (0.01, 0.07) 0.009	1.51 (0.57, 2.46) 0.002	0.97 (0.36, 1.58) 0.002	0.79 (0.14, 1.45) 0.019
Moderate/Severe Risk	0.10 (0.06, 0.13) < 0.001	0.30 (0.21, 0.39) < 0.001	0.09 (0.06, 0.12) < 0.001	2.45 (1.59, 3.31) < 0.001	1.45 (0.89, 2.01) < 0.001	1.60 (1.00, 2.20) < 0.001

Model 1 adjust for sex and age.

Model 2 adjust for sex, age, cholesterol, indirect bilirubin, glutamic pyruvic transaminase, creatinine, hemoglobin, fasting glucose.

The associations between malnourishment risk and morphological changes in the thoracic aorta were further examined in subgroups of study participants. The interaction between malnutrition risk and age concerning thoracic aorta morphological changes, including diameters and tortuosity, is or tends to be statistically significant. The β value of the association between GNRI and the diameter and tortuosity was stronger in older participants (≥65 years; Table 4).

**Table 4:** Subgroup analysis by age and sex.

**Statistics**	**Ascending and arcus aorta tortuosity**	**Thoracic aorta tortuosity**	**DTA tortuosity**	**L1 (mm)**	**L2 (mm)**	**L3 (mm)**
	β **(95% CI)** ***p*** **Value**	β **(95% CI)** ***p*** **Value**	β **(95% CI)** ***p*** **Value**	β **(95% CI)** ***p*** **Value**	β **(95% CI)** ***p*** **Value**	β **(95% CI)** ***p*** **Value**
<**65 years**						
1	Reference	Reference	Reference	Reference	Reference	Reference
2	0.00 (–0.05, 0.06) 0.851	0.08 (–0.03, 0.19) 0.175	0.03 (–0.00, 0.06) 0.063	0.96 (–0.23, 2.14) 0.117	0.59 (–0.13, 1.30) 0.112	0.31 (–0.53, 1.15) 0.475
3	0.11 (0.06, 0.17) < 0.001	0.18 (0.07, 0.29) 0.003	0.06 (0.03, 0.09) 0.004	1.89 (0.76, 3.03) 0.002	1.04 (0.35, 1.73) 0.004	0.86 (0.06, 1.67) 0.039
≥**65 years**						
1	Reference	Reference	Reference	Reference	Reference	Reference
2	0.02 (–0.04, 0.09) 0.465	0.14 (–0.03, 0.32) 0.115	0.06 (0.01, 0.12) 0.033	2.62 (0.94, 4.31) 0.003	1.68 (0.53, 2.84) 0.0055	1.52 (0.33, 2.71) 0.0145
3	0.13 (0.08, 0.18) < 0.001	0.47 (0.34, 0.61) < 0.001	0.12 (0.07, 0.17) < 0.001	2.83 (1.38, 4.27) < 0.001	1.76 (0.77, 2.75) < 0.001	2.20 (1.18, 3.22) < 0.001
*P* for interaction	0.086	0.005	0.018	0.081	0.085	0.015
**Male**						
1	Reference	Reference	Reference	Reference	Reference	Reference
2	0.03 (–0.01, 0.07) 0.118	0.11 (–0.00, 0.22) 0.061	0.04 (0.01, 0.07) 0.004	1.49 (0.49, 2.50) 0.004	1.22 (0.56, 1.88) < 0.001	0.83 (0.15, 1.51) 0.019
3	0.10 (0.06, 0.14) < 0.001	0.26 (0.13, 0.38) < 0.001	0.10 (0.07, 0.13) < 0.001	2.09 (1.08, 3.10) < 0.001	1.87 (1.21, 2.53) < 0.001	1.74 (1.06, 2.43) < 0.001
**Female**						
1	Reference	Reference	Reference	Reference	Reference	Reference
2	0.01 (–0.06, 0.09) 0.737	0.18 (–0.08, 0.43) 0.177	0.03 (–0.05, 0.10) 0.517	1.65 (–0.46, 3.77) 0.132	0.41 (–0.89, 1.71) 0.535	0.61 (–0.94, 2.15) 0.445
3	0.11 (0.06, 0.15) < 0.001	0.49 (0.33, 0.65) < 0.001	0.05 (–0.01, 0.11) 0.124	2.00 (0.26, 3.75) 0.028	0.17 (–0.89, 1.24) 0.750	0.67 (–0.61, 1.94) 0.309
*P* for interaction	0.073	0.054	0.895	0.221	0.442	0.985

1, absence of risk; 2, low risk; 3, moderate/severe risk.

## 4 Discussion

The number of cancer survivors is increasing exponentially due to advancements in early screening and treatment. However, all survivors continue to face significantly elevated risks of CVD (1, 21, 22). The incidence of malnutrition among cancer patients is notably high (23), and malnutrition has been established as a risk factor for adverse cardiovascular outcomes (8). Changes in aortic morphology, including alterations in diameter and tortuosity, influence vascular hemodynamics and contribute to cardiovascular outcomes (24). Nonetheless, the relationship between malnutrition and aortic morphology within the cancer population has not yet been reported. This study, conducted on cancer survivors, found that malnutrition, as assessed by the GNRI, was associated with an increased degree of thoracic aortic dilation and tortuosity, indicating a stiffening of aortic morphology. This association remained significant after adjusting for various potential confounders and was observed across all age groups and in males. These results suggest that nutritional status might influence vascular morphology and nutritional status warrants greater attention in clinical practice to monitor the progression of arterial stiffening in cancer patients. To the best of our knowledge, this is the first study to demonstrate the relationship between nutritional status and thoracic aortic morphology in cancer survivors.

Our subjects included patients with prevalent clinical cancers, such as lung cancer, gastrointestinal cancer, hematological cancer, and urogenital cancer. The prevalence of malnutrition among these subjects was 47.6%, which is consistent with the established prevalence of malnutrition in cancer patients (23). Malnutrition is linked to increased cardiovascular morbidity and mortality in patients with CVD, stroke, cancer, and chronic kidney disease (16, 25–29). A recent meta-analysis determined that malnutrition independently elevated all-cause mortality by 72% and major adverse cardiovascular events by 47% in patients with coronary artery disease (30). However, the relationship between malnutrition and alterations in cardiovascular morphology remains largely unknown. Aortic diameter and tortuosity have been recognized as age-related predictors of future CVD events (31, 32). The cardiovascular age of cancer patients is typically 6–8 years older than their chronological age, as determined by a risk factor prediction model (33). Thus, increased aortic diameter and tortuosity may indicate vascular aging. Our study found that cancer patients classified as being at moderate or severe risk of malnutrition exhibited larger thoracic aortic diameters and increased tortuosity of the thoracic aorta compared to those without such risk, which raises the question of whether accelerated cardiovascular aging due to malnutrition is a common factor which contributing to the poor prognosis of various diseases associated with malnutrition. This question merits further investigation.

Subgroup analysis revealed an interaction effect related to age, indicating that age moderated the association between the GNRI and the morphological remodeling of the thoracic aorta. Notably, individuals over 65 years with moderate-to-severe malnutrition risk exhibited greater diameter and tortuosity of the thoracic aorta compared to those without such risk in this exploratory analysis. Previous studies have documented progressive elongation and widening of the aorta with aging (11, 34). This finding may suggest that aging exacerbates the negative impact of malnutrition on the thoracic aorta. Additionally, aging is a significant risk factor for atherosclerosis (35), which is often accompanied by endothelial dysfunction, migration of smooth muscle cells, and vasomotor dysfunction (36, 37). The combined effects of aging and atherosclerosis might render the elderly aorta more susceptible to the adverse vascular consequences of malnutrition.

Elucidating the specific mechanisms by which malnutrition influences thoracic aortic tortuosity and dilation in cancer patients is challenging, given the results of this observational study. Elastic proteins in the middle layer of the aortic wall are crucial for maintaining vascular elasticity. The production of elastin is tightly regulated, occurring primarily in utero and during the first postnatal year; after this period, its synthesis ceases, resulting in a fixed quantity of elastin fibers throughout life (38–40). Age-related aortic stiffness is primarily attributed to the rupture and fragmentation of elastin fibers due to repeated stretching and contraction, which subsequently leads to aortic dilation and tortuosity (41). In animal studies, mice lacking elastin exhibit significantly longer aortas at birth, with varying levels of elastin influencing the rate of aortic lengthening (42). Malnutrition can result in inadequate protein synthesis and energy deficiency, leading to the gradual breakdown of elastin and other detrimental changes in the vascular walls. Furthermore, chronic low-grade inflammation, driven by cytokines IL-1β and TNF-α, along with oxidative stress, impacts extracellular matrix alterations, and aortic stiffness through MMP-mediated elastin degradation and TGF-β-associated collagen accumulation. Notably, malnutrition may exacerbate systemic inflammation in cancer patients (43, 44). Therefore, inflammation may represent another important factor linking malnutrition to alterations in aortic morphology. Our findings demonstrate a pronounced inverse correlation between GNRI and descending thoracic aorta tortuosity, which may reflect region-specific vulnerabilities to malnutrition-driven vascular remodeling. The DTA, unlike the ascending aorta, lacks vasa vasorum in its outer media, rendering it more dependent on luminal diffusion for nutrient supply. Malnutrition-induced hypoalbuminemia and oxidative stress likely impair endothelial nutrient transport, exacerbating medial hypoxia, and elastin fragmentation (40).

In contrast to the four classes proposed by Bouillanneet al. (13), we combined the moderate (GNRI: 82– < 92) and severe risk (GNRI: < 82) groups because the number of subjects with severe risk was very small in this study, which could lead to statistical error (9). This stratification aligns with ESPEN guidelines, where GNRI < 92 necessitates immediate nutritional intervention, while 92–98 warrants surveillance. In cardio-oncology contexts, GNRI < 92 independently predicts a 68% higher risk of aortic remodeling, underscoring its vascular relevance (45). While these GNRI categories were not cancer-specific, subsequent studies have adopted these cut-offs in oncology populations due to their robust correlation with clinical outcomes in cancer cohorts (46, 47). In addition, the GNRI was prioritized in our study due to its objectivity, reproducibility, and strong prognostic utility in oncology populations. Unlike tools requiring subjective patient-reported data (PG-SGA, which requires patient-generated symptom scores) or functional assessments (MNA, which assesses dietary intake and cognitive status), GNRI relies solely on serum albumin and body weight parameters routinely measured in clinical practice. This minimized missing data in our retrospective cohort. Additionally, GNRI has been validated as a predictor of morbidity and mortality in cancer patients across multiple studies (48). In a meta-analysis of 6,060 cancer patients, GNRI outperformed PG-SGA and MNA in predicting survival (49). While GNRI offers practical advantages in retrospective oncology research, it does not assess dietary intake, symptom burden, or body composition dimensions captured by PG-SGA or MNA. Future prospective studies should integrate multiple nutritional metrics to identify the most clinically informative tool for vascular risk stratification.

Several limitations were identified in our study. First, the sample size was relatively small, and the cross-sectional design limited our ability to establish causal relationships.

Despite the modest sample size, the observed effect sizes were clinically meaningful. For instance, GNRI < 92 was associated with an increase in severe aortic tortuosity, consistent with malnutrition's known role in collagen degradation and vascular structural changes. These findings, even if preliminary, highlight a plausible biological pathway warranting urgent investigation in larger cohorts. Second, our data analysis relied solely on the GNRI and measurements of thoracic aortic diameter and tortuosity at enrollment, without considering any changes in these values during long-term follow-up. The cross-sectional design prevents temporal assessment of GNRI changes relative to aortic tortuosity progression. While malnutrition may contribute to vascular remodeling, reverse causality cannot be ruled out. Thus, our findings should be interpreted as generating hypotheses for mechanistic and interventional studies, rather than supporting direct clinical action. Third, our analysis did not account for cancer stage-specific therapies, or inflammatory biomarkers, which may confound the GNRI-aortic tortuosity relationship. Future prospective studies should rigorously capture these variables to disentangle malnutrition-driven effects from tumor biology or treatment-related influences. We found no significant differences in tumor presence or type among the three GNRI categories. Fourth, the exclusion of patients with pre-existing hypertension, diabetes mellitus, or chronic kidney disease enhances internal validity by controlling for cardiometabolic confounders, this criterion may constrain the clinical applicability of findings to real-world oncology populations, where such comorbidities frequently coexist as part of multimorbidity patterns. Besides, the data in this study were derived from a retrospective cohort of cancer patients undergoing contrast-enhanced CT imaging with non-ECG-gated acquisition protocols, which may introduce potential measurement inaccuracies in assessing proximal aortic dimensions.

## 5 Conclusions

Our findings indicate that malnutrition measured by GNRI is linked to aortic diameter and tortuosity in cancer patients, reflecting the exploratory role in identifying malnutrition as a novel risk marker in cardio-oncology. Future studies could explore whether improving GNRI through targeted nutritional support mitigates aortic remodeling.

## Data Availability

The raw data supporting the conclusions of this article will be made available by the authors, without undue reservation.
