# Supplementary material for: Association between geriatric nutritional risk index and thoracic aorta morphological changes in cancer adults underwent contrast computed tomography scans
Source: Front Nutr. 2025 Aug 22;12:1583220. doi: 10.3389/fnut.2025.1583220 (PMC12411165; doi:10.3389/fnut.2025.1583220)
Supplement: Supplementary file 1 [file Table_1.docx]

**Table S1. The correlation coefficients of the GNRI with morphological characteristics of aorta**

| Variables | Correlation | 95% CI | P-value |
| --- | --- | --- | --- |
| Ascending and arcus aorta tortuosity | -0.544 | -0.641 to -0.427 | <0.001 |
| Thoracic aorta tortuosity | -0.530 | -0.636 to -0.405 | <0.001 |
| DTA tortuosity | -0.594 | -0.689 to -0.483 | <0.001 |
| L1,mm | -0.474 | -0.587 to -0.351 | <0.001 |
| L2,mm | -0.483 | -0.594 to -0.363 | <0.001 |
| L3,mm | -0.471 | -0.586 to -0.351 | <0.001 |

DTA: descending thoracic aorta; CI: confidence intervals.
